# Herb-Drug Interaction of *Paullinia cupana* (Guarana) Seed Extract on the Pharmacokinetics of Amiodarone in Rats

**DOI:** 10.1155/2012/428560

**Published:** 2012-12-05

**Authors:** Márcio Rodrigues, Gilberto Alves, Nulita Lourenço, Amílcar Falcão

**Affiliations:** ^1^Laboratory of Pharmacology, Faculty of Pharmacy, University of Coimbra, 3000-548 Coimbra, Portugal; ^2^Centre for Neuroscience and Cell Biology (CNC), University of Coimbra, 3004-517 Coimbra, Portugal; ^3^Health Sciences Research Centre (CICS), University of Beira Interior (UBI), 6200-506 Covilhã, Portugal; ^4^Department of Internal Medicine, Amato Lusitano Hospital, 6000-085 Castelo Branco, Portugal

## Abstract

*Paullinia cupana* is used in weight-loss programs as a constituent of medicinal/dietary supplements. This study aimed to assess a potential herb-drug interaction among a standardized (certified) *Paullinia cupana* extract and amiodarone (narrow therapeutic index drug) in rats. In a first pharmacokinetic study rats were simultaneously coadministered with a single dose of *Paullinia cupana* (821 mg/kg, p.o.) and amiodarone (50 mg/kg, p.o.), and in a second study rats were pretreated during 14 days with *Paullinia cupana* (821 mg/kg/day, p.o.) receiving amiodarone (50 mg/kg, p.o.) on the 15th day. Rats of the control groups received the corresponding volume of vehicle. Blood samples were collected at several time points after amiodarone dosing, and several tissues were harvested at the end of the experiments (24 h after dose). Plasma and tissue concentrations of amiodarone and its major metabolite (mono-*N*-desethylamiodarone) were measured and analysed. A significant reduction in the peak plasma concentration (73.2%) and in the extent of systemic exposure (57.8%) to amiodarone was found in rats simultaneously treated with *Paullinia cupana* and amiodarone; a decrease in tissue concentrations was also observed. This paper reports for the first time an herb-drug interaction between *Paullinia cupana* extract and amiodarone, which determined a great decrease on amiodarone bioavailability in rats.

## 1. Introduction

*Paullinia cupana*, commonly known as Guarana, is a climbing evergreen vine with small fruits and is native to the Amazon region [1, 2]. *Paullinia cupana* seed extracts have been used in folk medicine since pre-Columbian times as stimulants, aphrodisiacs, and tonics [2].

The seeds of* Paullinia cupana* contain large amounts of methylxanthines (caffeine, theophylline, and theobromin), saponins, and polyphenols, especially tannins, as well as trace concentrations of many other compounds [3–5]. These constituents are probably responsible for the use of *Paullinia cupana* seed extract in popular medicine as a stimulant of the central nervous system, in cases of physical and mental stress, and as an antidiarrheic, diuretic, and antineuralgic [5]. Its high caffeine content and stimulating properties make *Paullinia cupana* particularly attractive in weight-loss programs since it helps increase the metabolic rate and can improve thermogenesis. Indeed, *Paullinia cupana *(Guarana) containing products are popular among athletes because of their ergogenic and “fat burning” effects [6]. Some studies have shown that *Paullinia cupana* positively affects lipid metabolism [1], enhances weight loss [7, 8], and increases basal energy expenditure [4, 9]. Thus, these data suggest that *Paullinia cupana* has antiobesity effects [4, 7–9]. Moreover, several studies have also ascribed to *Paullinia cupana* antioxidant and cardioprotective effects [4, 6, 10, 11].

*Paullinia cupana* extract (from seed) is approved in the United States as a food additive and is considered a dietary supplement, [3]; *Paullinia cupana* is also listed in the official Brazilian Pharmacopoeia [12]. Therefore, it is not surprising that *Paullinia cupana* (Guarana) is found today in a wide variety of drinks, foods, dietary/herbal supplements, and pharmaceuticals [1, 12]. Consequently, perhaps concerned about the widespread use or potential abuse of *Paullinia cupana-*based products, the Committee on Herbal Medicinal Products (HMPC) of European Medicines Agency has recently launched a call to encourage the submission of scientific data on *Paullinia cupana* (Guarana) in order to prepare the corresponding herbal monograph [13].

Given that obesity and overweight are increasing at an alarming rate in developed countries [14], representing two of the major independent risk factors for cardiovascular diseases [15–17], a great increase on the consumption of dietary/herbal supplements containing *Paullinia cupana* is still expected. Since the concurrent use of these products with conventional drugs may lead to significant clinical herb-drug interactions, it is urgent to assess the interference of *Paullinia cupana *seed extract on the kinetics of narrow therapeutic index drugs, such as amiodarone [18].

**Figure 1:**
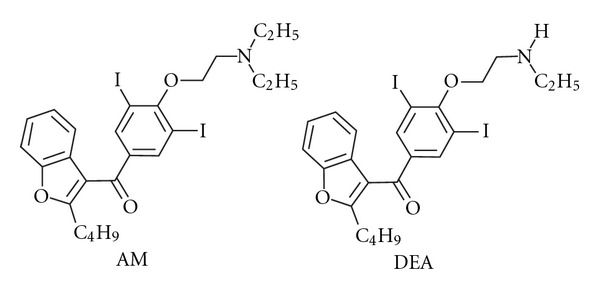
Chemical structures of amiodarone and its major metabolite mono-*N*-desethylamiodarone (MDEA).

Amiodarone [2-*n*-butyl-3-(3,5-diiodo-4-diethylaminoethoxy-benzoyl)-benzofuran; (Figure 1)] is one of the most widely prescribed antiarrhythmic agents for the treatment of atrial fibrillation and ventricular arrhythmias [19]. From a pharmacokinetic viewpoint amiodarone has unusual and complex properties [20, 21], and it is recognised as a drug of narrow serum/plasma therapeutic range (0.5–2.0 *μ*g/mL) [20, 22]. Furthermore, amiodarone has also been associated to important clinical drug interactions [23–25]. Nevertheless, to the best of our knowledge, no study was conducted until now to evaluate herb-drug interactions between *Paullinia cupana *(Guarana) and amiodarone.

Taking into account all the reasons previously referred and considering the high potential for the coadministration of *Paullinia cupana *medicinal products and amiodarone, this work was planned to investigate if a commercial standardized (certified) extract of *Paullinia cupana *seeds (see Supplementary Material available online at doi:10.1155/2012/428560) may influence the pharmacokinetics of amiodarone in rats following their simultaneous oral coadministration and after a 14-day* Paullinia cupana *pretreatment period.

## 2. Materials and Methods

### 2.1. Drugs and Materials

Guarana (*Paullinia cupana L.*) extract 12% caffeine obtained from fruit seeds was purchased from Bio Serae Laboratories (Bram, France); the corresponding certificate of analysis ref. 410044 (batch 0805519) is provided as a Supplementary Material. Carboxymethylcellulose sodium salt for the preparation of extract suspension was obtained from Sigma (St. Louis, MO, USA). A commercial formulation (ampoules) of amiodarone hydrochloride 50 mg/mL solution for the intravenous injection was used for oral administration to rats after appropriate dilution with 5% glucose intravenous solution for infusion (B. Braun Medical, Portugal). Other compounds used were sodium chloride 0.9% solution for injection (Labesfal, Portugal): heparin sodium 5000 U.I./mL for injection (B. Braun Medical, Portugal): ketamine for injection (Imalgene 1000), and xylazine for injection (Vetaxilaze 20). Introcan Certo IV indwelling cannula (22 G; 0.9 × 2.5 mm) is made of polyurethane from B. Braun Melsungen AG (Melsungen, Germany).

### 2.2. Animals

Adult male Wistar rats (310–380 g) of approximately 10 weeks old were obtained from local animal facilities (Faculty of Health Sciences of the University of Beira Interior, Covilhã, Portugal). The rats were maintained under controlled environmental conditions (temperature 20 ± 2°C; relative humidity 55 ± 5%; 12-h light/dark cycle). The animals were allowed free access to a standard rodent diet (4RF21, Mucedola, Italy) during almost all experimental procedures, and tap water was available *ad libitum*. At night on the day before dosing with amiodarone, a lateral tail vein of each rat was cannulated, under anaesthesia (ketamine (90 mg/kg)/xylazine (10 mg/kg); i.p. injection), by the insertion of an Introcan Certo IV indwelling cannula (22G; 0.9 × 2.5 mm) used for serial blood sampling. The rats were fully recovered from anaesthesia overnight and were fasted for 12–14 h before amiodarone administration and maintained with free access to water; to avoid the effect of food on the oral bioavailability of amiodarone an additional fasting period was considered (4 h post dose). Oral treatments of the rats with *Paullinia cupana *extract and amiodarone were performed by gavage. Blood sampling was conducted in conscious and freely moving rats, which were appropriately restrained only at the moment of blood collection, except for the last blood sampling that was taken by a terminal procedure (decapitation and exsanguination under anaesthesia). All the animal experiments were conducted in accordance with the European Directive (2010/63/EU) [26] for the accommodation and care of laboratory animals, and the experimental procedures were reviewed and approved by the Portuguese Veterinary General Division.

### 2.3. Experimental Design and Pharmacokinetic Studies

Two separate and independent pharmacokinetic studies were designed to investigate the effects of *Paullinia cupana *on the kinetics of amiodarone: (1) a single oral coadministration study of *Paullinia cupana *extract and amiodarone: and (2) a 14-day repeated oral pretreatment study with *Paullinia cupana *extract, and on the 15th day a single oral dose of amiodarone was given. The dose of *Paullinia cupana *was selected based on the dose recommended to humans by the supplier of the extract (Bio Serae Laboratories) and taking into account the Food and Drug Administration (FDA) Guidance for Industry on the conversion of animal doses to human equivalent doses [27]; additionally, a 10-fold potentiating interaction factor was considered. On the other hand, the single oral dose of amiodarone (50 mg/kg) was established because it has provided plasma concentrations of amiodarone in rats within the plasma therapeutic range [28]. In each day of the experiments *Paullinia cupana *extract was suspended in 0.5% carboxymethylcellulose aqueous solution affording a suspension of herbal extract at 82.1 mg/mL. Amiodarone commercial injectable solution (50 mg/mL) was also appropriately diluted with 5% glucose solution to extemporaneously prepare an amiodarone solution at 12.5 mg/mL. Appropriate volumes of *Paullinia cupana *extract suspension (10 mL/kg of body weight) and of amiodarone solution (4 mL/kg of body weight) were orally administered to rats by oral gavage.

In the first pharmacokinetic study, twelve Wistar rats were randomly divided into two groups (experimental and control groups). Rats of the experimental group (*n* = 6) were concomitantly treated with a single dose of *Paullinia cupana *extract (821 mg/kg, p.o.) and a single dose of amiodarone (50 mg/kg, p.o.); the extract suspension was administered right before amiodarone. Rats of the control group (*n* = 6) received, instead of the *Paullinia cupana *extract suspension, the corresponding volume of 0.5% carboxymethylcellulose aqueous solution (vehicle of the extract).

In the second pharmacokinetic study, twelve Wistar rats were also randomly divided into two groups. Rats assigned to the experimental group (*n* = 6) were orally pretreated with *Paullinia cupana *extract (821 mg/kg, p.o.) once daily for 14 consecutive days (subchronic pretreatment). Rats allocated to the control group (*n* = 6) were administered with an equivalent volume of vehicle for the same period of time. During the pretreatment period, the rats were kept in 12 h light/dark cycle animal room with controlled temperature and humidity, as indicated above (see Section 2.2.); free access to a standard rodent diet and tap water was allowed. On 15th day, rats of both groups (experimental and control) were gavaged with a single dose of amiodarone (50 mg/kg, p.o.).

In both pharmacokinetic studies, the treatments with *Paullinia cupana *extract (or vehicle) and/or amiodarone were always carried out on the morning between 9:00 AM and 11:45 AM. At night on the day before amiodarone administration, the rats were anaesthetized for cannulation of a lateral tail vein and were fasted overnight as described in the previous section. On the day after, multiple serial blood samples (approximately 0.3 mL) were collected through the cannula into heparinized tubes before dosing and at 0.25, 0.5, 1, 2, 4, 6, 8, and 12 h following amiodarone administration; then, at 24 h after dose, blood and tissues (heart, liver, kidneys, and lungs) were also harvested after the decapitation of the rats. The blood samples were centrifuged at 4000 rpm for 10 min (4°C) to separate the plasma which was stored at −20°C until analysis. After exsanguination, liver, kidneys, heart, and lungs were excised and stored at −20°C; the organs were weighed and homogenized in distilled water (3 mL of water per gram of tissue) before analysis of tissue homogenates samples.

### 2.4. Drug Analysis

Plasma and tissue concentrations of amiodarone and its main metabolite [mono-*N*-desethylamiodarone (MDEA)] were determined by using a liquid-liquid extraction (LLE) procedure followed by high-performance liquid chromatography-diode array detection (HPLC-DAD) assay previously developed and validated [29]. Briefly, an aliquot of each plasma sample (150 *μ*L) was diluted with 150 *μ*L of 0.1 M sodium phosphate buffer (pH 5) and spiked with 20 *μ*L of the IS working solution (50 *μ*g/mL). The mixture was added 500 *μ*L of *n*-hexane (used as LLE solvent), vortex mixed for 30 sec and centrifuged at 17000 rpm for 2 min at 4°C. The upper organic layer was transferred to a clean glass tube, and the sample was reextracted two more times with* n*-hexane (500 *μ*L each time) using the same experimental conditions. Then, the whole organic extract was evaporated to dryness under a nitrogen stream at 60°C, and the residue was reconstituted in 100 *μ*L of methanol. Following this, an aliquot of the reconstituted extract (20 *μ*L) was injected into the HPLC system for analysis.

For the extraction from tissues, each aliquot (400 *μ*L) of tissue (heart, liver, kidney, and lung) homogenates was spiked with 20 *μ*L of the IS working solution (50 *μ*g/mL); then the mixture was added 400 *μ*L of acetonitrile (used as protein precipitating agent), vortex mixed for 1 min and centrifuged at 17000 rpm for 10 min at 4°C in order to precipitate the protein content. The supernatant was transferred to a new propylene tube, and 1 mL of *n*-hexane (used as LLE solvent) was added. The mixture was vortex mixed for 1 min and centrifuged at 17000 rpm for 5 min at 4°C. The upper organic layer (*n*-hexane) was transferred to a clean glass tube, and the sample was reextracted two more times with *n*-hexane (0.8 mL each time) using the same conditions. The organic extract was evaporated to dryness, reconstituted, and then injected into the HPLC system using the same procedures as mentioned above for rat plasma samples. The limit of quantification (LOQ) was established at 0.100 *μ*g/mL for amiodarone and MDEA in plasma and in tissue homogenates.

### 2.5. Pharmacokinetic Analysis

The plasma concentration *versus* time data for amiodarone and MDEA obtained from each individual rat were submitted to a noncompartmental pharmacokinetic analysis using the WinNonlin version 4.1 (Pharsight Co, Mountain View, CA, USA). The peak concentrations of amiodarone and MDEA in plasma (*C*_max⁡_) and the time to reach *C*_max⁡_ (*t*_max⁡_) were obtained directly from the experimental data. Other pharmacokinetic parameters estimated from the individual plasma concentration-time profiles were area under the concentration-time curve (AUC) from time zero to the last sampling time at which concentrations were at or above the LOQ of the method (AUC_0–*t*_), calculated by the linear trapezoidal rule; AUC from time zero to infinite (AUC_0–∞_), calculated from AUC_0–*t*_ + (*C*_last_/*k*_el_), where *C*_last_ is the last quantifiable concentration and *k*_el_ is the apparent terminal rate constant calculated by log-linear regression of the terminal segment of the concentration-time profile; apparent terminal elimination half-life (*t*_1/2el_) and mean residence time (MRT). The concentrations lower than the LOQ of the assay were taken as zero for all calculations.

### 2.6. Effect of the Subchronic *Paullinia cupana* Treatment on Body Weight

For the subchronic treatment study (a 14-day* Paullinia cupana *treatment period), the body weight of the rats administered with *Paullinia cupana *extract (821 mg/kg/day, p.o.; experimental group) or vehicle (control group) was adequately registered on the first day and on the last day (14th) of these treatments in order to examine the effect of *Paullinia cupana *extract on body weight changes.

### 2.7. Statistical Analysis

Data were reported as the mean ± standard error of the mean (SEM). Comparisons between two groups were usually performed using unpaired two-tailed Student's *t*-test; for body weight comparisons within the same group the paired Student's *t*-test was employed. A difference was considered to be statistically significant for a *P* value lower than 0.05 (*P* < 0.05).

## 3. Results

### 3.1. Effects of the Simultaneous Coadministration of *Paullinia cupana* on Amiodarone Pharmacokinetics

**Figure 2:**
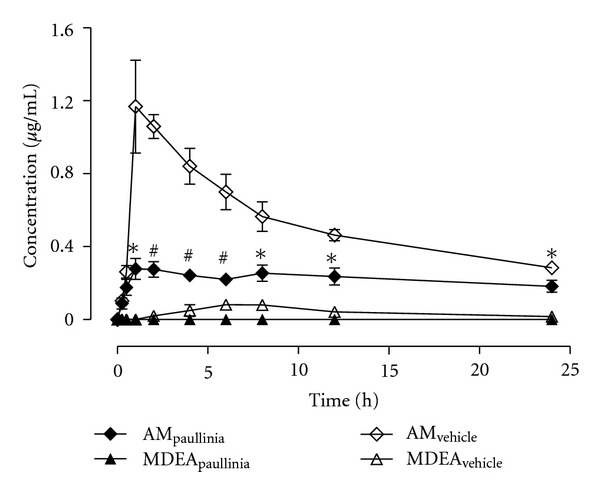
Mean plasma concentration-time profiles of amiodarone and mono-*N*-desethylamiodarone (MDEA) obtained, over a period of 24 h, from rats simultaneously treated in single dose with *Paullinia cupana *extract (821 mg/kg, p.o.), or vehicle (0.5% carboxymethylcellulose aqueous solution), and amiodarone (50 mg/kg, p.o.) by oral gavage. Symbols represent the mean values ± standard error of the mean (SEM) of six determinations per time point (*n* = 6). *P < 0.05 and ^#^ *P* < 0.001 compared to control (vehicle).

**Figure 3:**
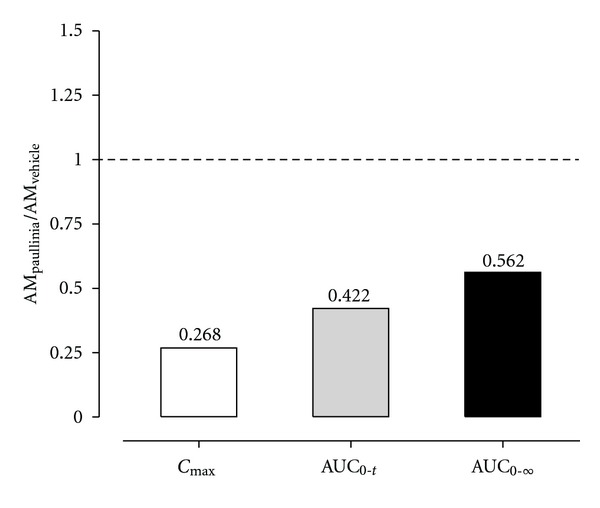
Ratios for the main plasma pharmacokinetic parameters (*C*_max⁡_, AUC_0–*t*_ and AUC_0–∞_) estimated for amiodarone in rats simultaneously treated in single dose with *Paullinia cupana *extract (821 mg/kg, p.o.), or vehicle (0.5% carboxymethylcellulose aqueous solution), and amiodarone (50 mg/kg, p.o.) by oral gavage.

The mean plasma concentration-time profiles (*n* = 6) of amiodarone and its main metabolite (MDEA) obtained after intragastric coadministration of rats with a single dose of *Paullinia cupana *extract (821 mg/kg, p.o.) or vehicle (control group) and a single dose of amiodarone (50 mg/kg, p.o.) are shown in Figure 2. Amiodarone plasma concentrations were similar in both groups only at the first two time-points after dose (up to 0.5 h). Amiodarone plasma concentrations in the group treated with *Paullinia cupana *extract were significantly lower than those in the control group over the 1–24 h post dose time period (at least, *P* < 0.05). For MDEA, the plasma concentrations were only detected (not quantified) in the rats treated with *Paullinia cupana* extract, and in the rats of the control group the plasma concentrations were also low with values near or below the LOQ (0.100 *μ*g/mL). The main plasma pharmacokinetic parameters estimated for amiodarone and MDEA after noncompartmental analysis of their concentration-time profiles are summarized in Table 1. With the coadministration of *Paullinia cupana *extract the mean *C*_max⁡_ of amiodarone was significantly lower than that obtained in the control (vehicle) group (*P* < 0.001), while the mean time to reach *C*_max⁡_ (*t*_max⁡_) was attained later in the experimental group (3.67 ± 1.17 h) comparatively to the control group (1.83 ± 0.48 h). Statistically significant differences were also observed for the AUC_0–*t*_ pharmacokinetic parameter (*P* < 0.001) calculated from the plasma concentration-time data obtained for amiodarone in both groups; these differences were also evident for the AUC_0–∞_ parameter (*P* < 0.05) (Table 1). Taking into consideration the information derived from Figure 3, it is clear that following the simultaneous coadministration of *Paullinia cupana *extract and amiodarone a remarkable decrease (73.2%) in the *C*_max⁡_ of the drug was observed, as well as a reduction of 57.8% in the extent of systemic drug exposure (as assessed by AUC_0–*t*_). The mean values estimated for the elimination pharmacokinetic parameters are similar in both groups (*Paullinia cupana *extract *versus* vehicle). Considering the paucity of quantifiable plasma concentrations obtained for MDEA, it was only possible to present the *C*_max⁡_ and *t*_max⁡_ parameters to the control (vehicle) group (Table 1).

**Figure 4:**
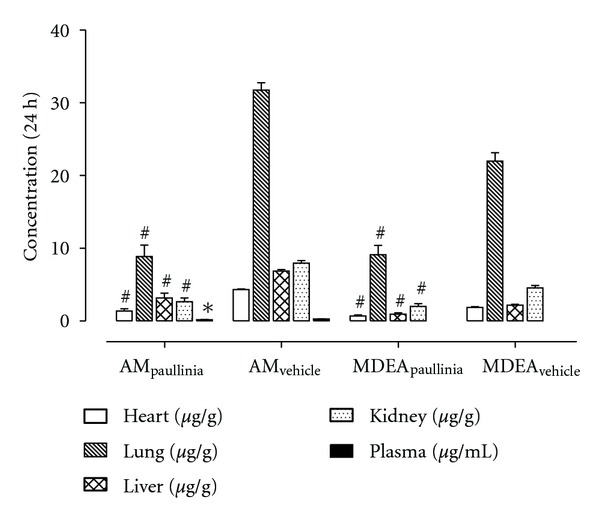
Mean plasma and tissue (heart, lung, liver, and kidney) concentrations of amiodarone and mono-*N*-desethylamiodarone (MDEA) obtained, at 24 h after dose, from rats simultaneously treated in single dose with *Paullinia cupana *extract (821 mg/kg, p.o.), or vehicle (0.5% carboxymethylcellulose aqueous solution), and amiodarone (50 mg/kg, p.o.) by oral gavage. Data are expressed as the mean values ± standard error of the mean (SEM) of six determinations (*n* = 6). *P < 0.05 and ^#^ *P* < 0.001 compared to control (vehicle).

In addition, to examine some aspects related to the biodistribution of amiodarone and MDEA in rats, both in the presence or absence of the coadministration with *Paullinia cupana*, the animals were sacrificed at 24 h after dosing, and several tissues were excised and analysed. The mean concentrations of amiodarone and MDEA determined in heart, lung, liver, and kidney tissues, and also their plasma concentrations at the same time (24 h) are shown in Figure 4. As indicated in Figure 4, the tissue concentrations of amiodarone and MDEA were markedly higher than those determined in plasma and were absolutely noteworthy the levels found for both compounds (amiodarone and MDEA) in the lung tissue. In addition, significant differences were found in the concentrations of amiodarone and MDEA in tissues (heart, liver, kidney, and lung) collected from experimental (*Paullinia cupana *extract) and control (vehicle) groups (*P* < 0.001) at 24 h after dose. As expected, the tissue concentrations in the group treated with *Paullinia cupana *were lower than those measured in the control group, reflecting the differences observed in the extent of systemic drug exposure.

### 3.2. Effects of the Subchronic Pretreatment with *Paullinia cupana* on Amiodarone Pharmacokinetics

**Figure 5:**
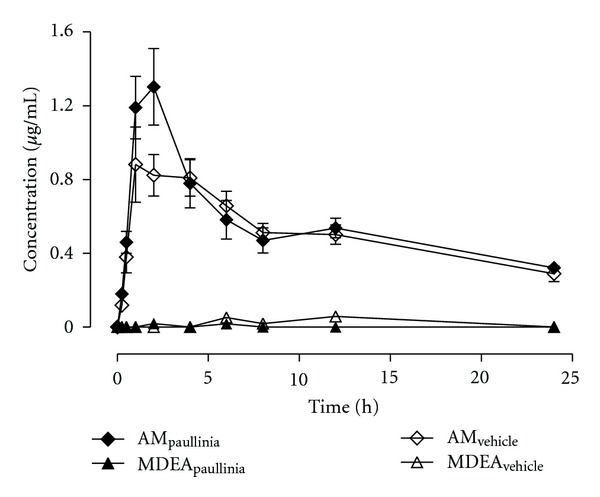
Mean plasma concentration-time profiles of amiodarone and mono-*N*-desethylamiodarone (MDEA) obtained, over a period of 24 h, from rats submitted to a 14-day pretreatment period with *Paullinia cupana *extract (821 mg/kg/day, p.o.), or vehicle (0.5% carboxymethylcellulose aqueous solution), and treated on the 15th day with a single dose of amiodarone (50 mg/kg, p.o.) by oral gavage (*n* = 6). Symbols represent the mean values ± standard error of the mean (SEM) of six determinations per time point (*n* = 6).

**Figure 6:**
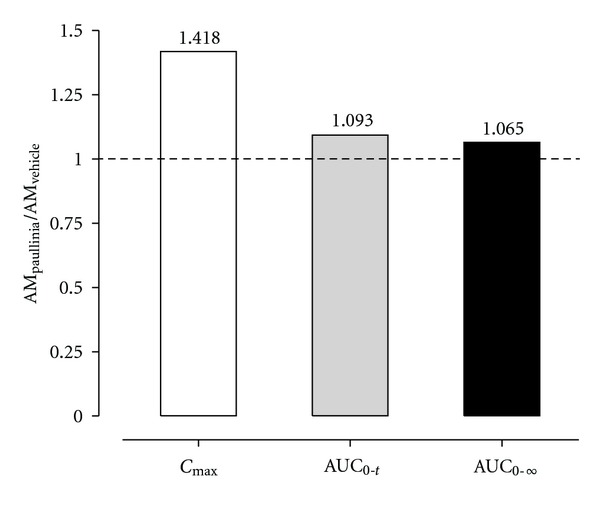
Ratios for the main plasma pharmacokinetic parameters (*C*_max⁡_, AUC_0–*t*_ and AUC_0–∞_) estimated for amiodarone in rats submitted to a 14-day pretreatment period with *Paullinia cupana *extract (821 mg/kg/day, p.o.), or vehicle (0.5% carboxymethylcellulose aqueous solution), and treated on the 15th day with a single dose of amiodarone (50 mg/kg, p.o.) by oral gavage.

The rats were administered for 14 days with *Paullinia cupana *extract (821 mg/kg, p.o.) or vehicle (control group) in order to investigate a possible interference of the* Paullinia cupana *subchronic treatment on the pharmacokinetics of amiodarone. The animals were administered with 50 mg/kg amiodarone (p.o.) one day after the last treatment with *Paullinia cupana *extract or vehicle, and the mean plasma concentration-time profiles (*n* = 6) of amiodarone and its main metabolite (MDEA) are depicted in Figure 5. The corresponding pharmacokinetic parameters, calculated by using noncompartmental analysis, are listed in Table 2. Overall, a close overlap was observed between the amiodarone plasma pharmacokinetic profiles at the early absorption phase (up to 0.5 h) and at the elimination phase (4–24 h). Amiodarone plasma concentrations in the group treated with *Paullinia cupana* were higher than those in the control group over the 1-2 h post dose time period (Figure 5). However, no significant differences (*P* > 0.05) in the pharmacokinetic parameters were detected for amiodarone and its main metabolite (MDEA) among the two groups (*Paullinia cupana versus* vehicle pretreatment) (Table 2). The plasma concentrations of MDEA were near or below the LOQ (0.100 *μ*g/mL) of the method in both groups of rats. Regarding the data shown in Figure 6, it appears that the rate of systemic exposure (as assessed by *C*_max⁡_) is slightly higher in the rats pretreated with *Paullinia cupana* extract, whereas the extent of systemic exposure to amiodarone is similar among experimental and control groups (ratios near to unity, as assessed by AUC_0–*t*_ and AUC_0–∞_).

**Figure 7:**
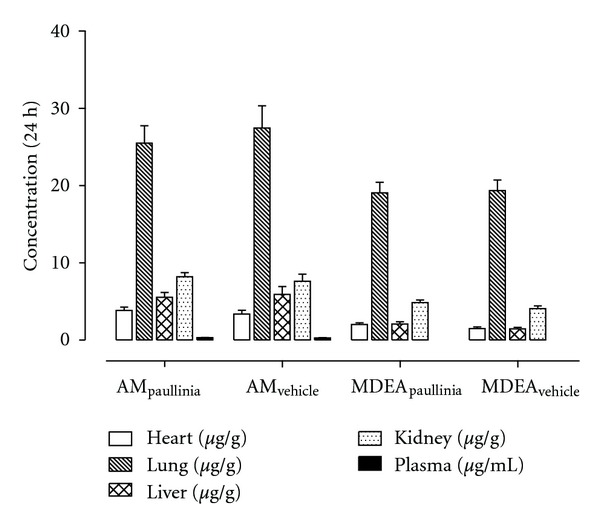
Mean plasma and tissue (heart, lung, liver and kidney) concentrations of amiodarone and mono-*N*-desethylamiodarone (MDEA) obtained, at 24 h after dose, from rats submitted to a 14-day pretreatment period with *Paullinia cupana *extract (821 mg/kg/day, p.o.), or vehicle (0.5% carboxymethylcellulose aqueous solution), and treated on the 15th day with a single dose of amiodarone (50 mg/kg, p.o.) by oral gavage. Data are expressed as the mean values ± standard error of the mean (SEM) of six determinations (*n* = 6).

To examine the influence of a 14-day pretreatment period with *Paullinia cupana *extract (experimental group) or vehicle (control group) on the distribution and metabolism of amiodarone in rats, the concentrations of amiodarone and its major metabolite (MDEA) were also determined in various tissues (additionally to plasma) at 24 h after dose, and the data are shown in Figure 7. As pointed out in Figure 7, the concentrations of both compounds (amiodarone and MDEA) in tissues were distinctly greater than those measured in plasma, and the concentration levels found in lung tissue were extremely high in experimental (*Paullinia cupana*) and control (vehicle) groups. As it was expected from the plasma/systemic pharmacokinetic profiles obtained, no significant differences were detected in tissue concentrations between both groups.

### 3.3. Effect of the Subchronic *Paullinia cupana* Treatment on Body Weight

**Figure 8:**
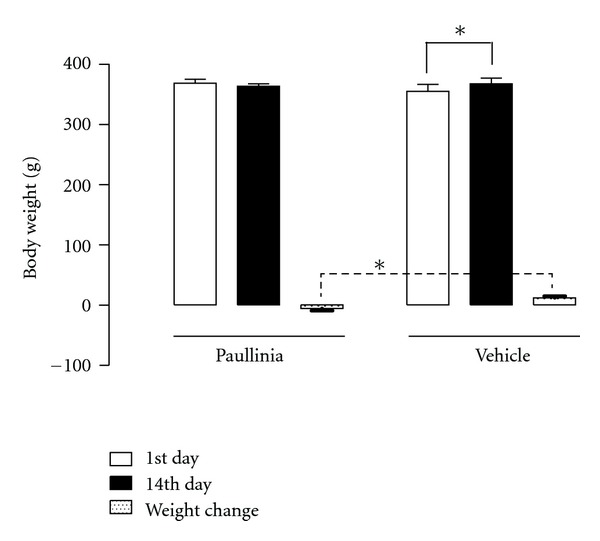
Effects on the body weight of the rats induced by the subchronic treatment (14-day period) with *Paullinia cupana* extract (821 mg/kg/day, p.o.) and vehicle (0.5% carboxymethylcellulose aqueous solution) by oral gavage (*n* = 6). *P < 0.01.

The resulting changes in body weight of the rats submitted to a 14-day treatment period with *Paullinia cupana *extract (821 mg/kg/day, p.o) or vehicle are demonstrated in Figure 8. From the analysis of the data it was found an increase statistically significant in body weight of rats treated with vehicle (control group) between the 1st and 14th day (*P* < 0.01), but differences were not observed in the group of rats administered with *Paullinia cupana *extract (*P* > 0.05). On the other hand, by comparing the weight changes in both groups (*Paullinia cupana *extract *versus* vehicle) a statistically significant difference was found, showing that the treatment with *Paullinia cupana *extract was able to suppress gains in body weight of rats.

## 4. Discussion

In literature, some interactions have been reported describing the interference of compounds, including herbal products, on the pharmacokinetics of amiodarone. Between them, grapefruit juice can inhibit dramatically amiodarone metabolism [30], in addition, orlistat can reduce significantly the systemic exposure to amiodarone and MDEA [31], and, more recently, the exposure of rats to *β*-naphthoflavone (a polycyclic aromatic hydrocarbon) was found to increase the formation of MDEA probably through cytochrome P450 (CYP) induction [32]. The present work was designed to investigate *in vivo* the potential of interaction between *Paullinia cupana *extract and amiodarone, using adult male Wistar rats (a whole-animal model). Taking into account that drug-drug or herb-drug interactions mainly occur at the level of absorption and/or metabolic (inhibition or induction) pathways, the pharmacokinetic studies reported herein were designed to examine the interference of *Paullinia cupana *extract on the gastrointestinal absorption (simultaneous coadministration study) and on the metabolism of amiodarone (14-day *Paullinia cupana *pretreatment study).

Our results clearly evidenced a significant decrease (73.2%) in the peak plasma concentration (*C*_max⁡_) of the drug following the simultaneous coadministration of the *Paullinia cupana *extract and amiodarone, as well as a reduction of 57.8% in the extent of systemic drug exposure (as assessed by AUC_0–*t*_). On the other hand, an increase was also observed on the rate of systemic exposure to amiodarone (as assessed by *C*_max⁡_) while no important differences were found in the extent of systemic drug exposure (as assessed by AUC_0–*t*_ and AUC_0–∞_) after the administration of the drug to pretreated rats one day after the last treatment with *Paullinia cupana *extract or vehicle. Hence, it is apparent that *Paullinia cupana *extract or its components interact with amiodarone in the gastrointestinal tract, reducing significantly the bioavailability of the drug after their simultaneous coadministration in a single dose. However, the similarity observed in the extent of systemic exposure to amiodarone in rats pretreated for 14 days with *Paullinia cupana *extract or vehicle excludes the impact of *Paullinia cupana*-induced metabolism on the bioavailability of the drug. In fact, in the rats pretreated for 14 days with *Paullinia cupana *extract an increasing of the rate of systemic exposure to amiodarone was apparent, but this difference was not statistically significant. Thus, the concomitant administration of *Paullinia cupana* extract and amiodarone in a single dose study supports the significance of the interaction at the level of the gastrointestinal tract. A significant reduction of the absorption of amiodarone induced by orlistat in healthy volunteers was also observed [31]; this drug, a lipase inhibitor, significantly reduced the systemic exposure to amiodarone by approximately 25%, and a decrease of similar magnitude (~25%) was detected in the generation of the metabolite MDEA (the major metabolite of amiodarone). According to Zhi et al. [31] the absorption of highly lipophilic drugs such as amiodarone may depend on the presence of a lipid phase in the gastrointestinal environment, which may be affected by the pharmacological action of orlistat. The rate (*C*_max⁡_) and extent (AUC_0–*t*_) of absorption of amiodarone were also enhanced in healthy volunteers who received a single dose of the drug immediately after consuming a high-fat meal *versus* following an overnight fast [33]. Shayengapour et al. [28] also studied the effects of food on the pharmacokinetics of amiodarone in rats. The results obtained concerning the interference of lipids on the oral bioavailability of amiodarone corroborated those reported in humans.

Taking into account the great diversity of components present in *Paullinia cupana *extract, especially methylxanthines (caffeine) and large quantity of tannins [34], we hypothesize the occurrence of a physical-chemical interaction between those compounds and amiodarone in the gastrointestinal tract of rats to explain the considerable decrease in the systemic exposure/bioavailability of amiodarone observed after the simultaneously coadministration with *Paullinia cupana *extract. However, further studies are needed to understand the mechanism associated to this herb-drug interaction (*Paullinia cupana *extract/amiodarone), which is reported herein for the first time. 

The central role that CYPs induction or inhibition play on drug-drug and herb-drug interactions is well recognised. Hence, to check the possible interference of *Paullinia cupana *extract on the CYP activity, the extract was administered for 14 days (821 mg/kg/day, p.o.) until 24 h before applying amiodarone; however, only an apparent influence was observed on the rate of systemic (plasma) exposure to amiodarone in these circumstances. Although this finding has not had in this case a significant impact on the magnitude of systemic exposure to amiodarone, it is an interesting aspect to explore in further studies directed to evaluate the potential of enzyme induction or inhibition by *Paullinia cupana*.

Globally, considering the rat plasma data generated in the present work and that reported from clinical studies following oral administration of amiodarone, it is clear that MDEA is the major metabolite of amiodarone in both species, despite differences will exist in their metabolite-to-parent ratios. Indeed, in our pharmacokinetic studies, the plasma concentrations of MDEA found in rat were significantly lower than those of amiodarone and were found at levels near or below the LOQ (0.100 *μ*g/mL) of the bioanalytical assay. Furthermore, amiodarone and MDEA were found in concentrations considerably lower in plasma than in tissues (heart, liver, lungs, and kidneys) at 24 h after dose, supporting their great plasma/tissue distribution; these differences were absolutely remarkable for plasma/lung tissue. These rat tissues were selected for bioanalysis of amiodarone and MDEA because they represent important targets from therapeutic (heart), toxicological (liver and lungs), and pharmacokinetic (liver and kidneys) viewpoints.

Based on data of herb-drug interaction between *Paullinia cupana *extract and amiodarone derived from this nonclinical investigation in rat, it is suggested that patients who are taking amiodarone should avoid the concurrent administration of herbal medicines/supplements containing *Paullinia cupana.* However, taking also into account the absence of a significant herb-drug interaction between the extract and amiodarone in the 14-day *Paullinia cupana *pretreatment study, an important impact on the drug efficacy is not expected if medicinal products containing *Paullinia cupana *extract and amiodarone are administered separately in the time. It is also true that results from animal experiments cannot be directly extrapolated to humans; however, bearing in mind the studies of Shayeganpour et al. [28] and Meng et al. [33] the rat appears to be an appropriate animal model for man in this situation. Nevertheless, to reliably assess the clinical outcomes of the interaction between *Paullinia cupana *extract and amiodarone-specific clinical trials are needed. 

## 5. Conclusions

In conclusion, to the best of our knowledge, this work is the first to document the interaction of *Paullinia cupana *with amiodarone. The decrease in amiodarone plasma concentrations was also accomplished by a significant reduction in the tissue concentrations of amiodarone and MDEA, particularly in the heart (therapeutic target organ or biophase). Hence, considering the available information, it is prudent not to take concomitantly amiodarone and medicinal products/dietary supplements containing *Paullinia cupana* despite our data support some potential of *Paullinia cupana *extract to weight management.

## Supplementary Material

ppCertificate of analysis of Guarana (*Paullinia cupana L.*) extract 12% caffeine provided by Bio Serae Laboratories (ref. 410044 - batch 0805519).

## Figures and Tables

**Table 1:** Pharmacokinetic parameters estimated by noncompartmental analysis of the plasma concentration-time profiles of amiodarone (AM) and mono-*N*-desethylamiodarone (MDEA, major metabolite of AM) obtained in rats after the simultaneous coadministration in single dose of *Paullinia cupana* extract (821 mg/kg, p.o.), or vehicle (0.5% carboxymethylcellulose aqueous solution), with AM (50 mg/kg, p.o.) by oral gavage (*n* = 6, unless otherwise noted).

Parameter	AM_Paullinia_		AM_Vehicle_	
	AM	MDEA	AM	MDEA
*t*_max⁡_ (h)	3.67 ± 1.17	NA	1.83 ± 0.48	7.20 ± 1.36^a^
*C*_max⁡_ (*μ*g/mL)	0.370 ± 0.043*	NA	1.378 ± 0.179	0.125 ± 0.012^a^
AUC_0–*t *_ (*μ*g·h/mL)	5.387 ± 0.619*	ND	12.774 ± 0.688	ND
AUC_0–∞_ (*μ*g·h/mL)	12.050 ± 2.118^#^	ND	21.431 ± 2.077	ND
*k*_el_ (h^−1^)	0.0310 ± 0.0044	ND	0.0433 ± 0.0082	ND
*t*_1/2el_ (h)	24.18 ± 2.75	ND	20.73 ± 5.74	ND
MRT (h)	36.87 ± 4.28	ND	28.64 ± 7.74	ND

NA: not available.

ND: not determined.

^a^ *n* = 5.

*P < 0.001, significantly different from the control group.

^#^ *P* < 0.05, significantly different from the control group.

**Table 2:** Pharmacokinetic parameters estimated by noncompartmental analysis of the plasma concentration-time profiles of amiodarone (AM) and mono-*N*-desethylamiodarone (MDEA, major metabolite of AM) obtained in rats submitted to a 14-day pretreatment period with *Paullinia cupana *extract (821 mg/kg/day, p.o.), or vehicle (0.5% carboxymethylcellulose aqueous solution), and treated on the 15th day with a single dose of AM (50 mg/kg, p.o.) by oral gavage (*n* = 6, unless otherwise noted).

Parameter	AM_Paullinia_		AM_Vehicle_	
	AM	MDEA	AM	MDEA
*t*_max⁡_ (h)	1.50 ± 0.22	2.00 ± 0.00^a^	2.00 ± 0.63	9.00 ± 2.12^b^
*C*_max⁡_ (*μ*g/mL)	1.483 ± 0.203	0.112 ± 0.00^a^	1.046 ± 0.151	0.106 ± 0.004^b^
AUC_0–*t *_ (*μ*g·h/mL)	13.422 ± 1.266	ND	12.282 ± 1.047	ND
AUC_0–∞_ (*μ*g·h/mL)	23.489 ± 1.970	ND	22.057 ± 2.905	ND
*K*_el_ (h^−1^)	0.0416 ± 0.0079	ND	0.0442 ± 0.0108	ND
*t*_1/2el_ (h)	23.07 ± 7.58	ND	21.62 ± 5.85	ND
MRT (h)	32.65 ± 10.41	ND	31.02 ± 8.29	ND

ND: not determined.

^a^ *n* = 1; ^b^ *n* = 4.
