# Supplementary material for: Herb-Drug Interaction of Paullinia cupana (Guarana) Seed Extract on the Pharmacokinetics of Amiodarone in Rats
Source: Evid Based Complement Alternat Med. 2012 Dec 5;2012:428560. doi: 10.1155/2012/428560 (PMC3523151; doi:10.1155/2012/428560)
Supplement: Supplementary file 1 — ppCertificate of analysis of Guarana (Paullinia cupana L.) extract 12% caffeine provided by Bio Serae Laboratories (ref. 410044 - batch 0805519). [file 428560.f1.pdf]

Bio serae
Laboratoires S.A.S.

GUARANA EXTRACT 12%

Paullinia cupana

CERTIFICATE OF ANALYSIS
Ref. 410044 - Batch 0805519

	Methods	Specifications	Results
**MAIN CHARACTERISTICS**			
Appearance	Bio Serae	free flowing powder	complies
Color	Bio Serae	light brown	complies
Carrier	Bio Serae	maltodextrin	complies
Nature	Bio Serae	alcoholic (30%) extract from seeds	complies
Odor	Bio Serae	slight	complies
Taste	Bio Serae	bitter	complies
**ACTIVE INGREDIENTS**			
Caffeine (%/RM)	HPLC - UV	≥ 12	12,7
**MICROBIOLOGICAL**			
Escherichia coli (CFU/10g)	Eur. Ph. 2000 [§2.6.13]	negative	negative
Total plate count (CFU/g)	NF ISO 4833	< 5 000	< 10
Yeasts and moulds (CFU/g)	AOAC 997.02	< 100	< 10
Salmonella (CFU/25g)	AES 10/04-05/04	negative	negative
Staphylococcus aureus (DNAse +) (CFU/10g)	Eur. Ph. 2000 [§2.6.13]	negative	negative
**OTHER ANALYTICAL DATA**			
Ashes (%/RM)	JORF (8 sept. 77)	< 2	0,49
Bulk density	Eur. Ph.1997 (§ 2.9.15)	> 0,30	0,66
Dry matter (%)	Eur.Ph.1997(§2.2.32 - 105°C)	> 90	95,3
Water solubility (10g/L)	NF EN 872 - GFC adapted	soluble	complies
**CONTAMINANTS**			
Cadmium (ppm)	Mineralization + ICP-MS	< 0,5	< 0,002
Heavy metals (lead eq.) (ppm)	Eur. Ph. 2000 [§2,4,8D]	< 15	< 10
GMO status	1829-1830/2003CE	Conventional ingredient	complies
Lead (ppm)	Mineralization + ICP-MS	< 1	< 0,03

STORAGE

24 months If kept in a dry and cool place in original intact packing

Manufacturing / analysis date : 04 / 2008

Best before : 04 / 2010

The information here above is based on our current knowledge. BIO SERAE cannot be hold responsible besides the guarantees written on its supply contracts, based on the fact that it does not control the final use of this product. It is the buyer's responsibility to comply with local texts and laws regulating its activity and the use of this product.

CFU = Colony-Forming Unit
DM = Dry Matter
RM = Raw Matter

Bram, 02-sept-08

Qualified batch

Mr. Murat
Quality Control Department

TOTALLY CHLORINE FREE

Siège social : N°1 avenue de la Preuilhe • Parc Technologique du Lauragais • 11150 Bram • FRANCE
Tel : 33 (0)4 68 76 76 20 • Fax : 33 (0)4 68 76 76 21 • info@bioserae.com • www.bioserae.com
S.A.S au Capital de 596511 euros • R.C.S. Carcassonne 329 581 375 • Code APE : 1089Z • N° T.V.A. : FR 51 329 581 375
